# Mortality Predictors and Associated Factors in Patients in the Intensive Care Unit: A Cross-Sectional Study

**DOI:** 10.1155/2020/1483827

**Published:** 2020-08-01

**Authors:** Fernanda G. de M. Soares Pinheiro, Eduesley Santana Santos, Íkaro Daniel de C. Barreto, Carleara Weiss, Andreia C. Vaez, Jussiely C. Oliveira, Matheus S. Melo, Francilene A. Silva

**Affiliations:** ^1^Nursing Department, Federal University of Sergipe, Lagarto, Sergipe, Brazil; ^2^Graduate Program in Nursing, Federal University of Sergipe, São Cristóvão, Sergipe, Brazil; ^3^Graduate Program of Biometrics and Applied Statistics, Federal Rural University of Pernambuco, Recife, Brazil; ^4^Jacobs School of Medicine and Biomedical Sciences, University at Buffalo, Buffalo, NY, USA; ^5^Nursing Department, Federal University of Sergipe, São Cristóvão, Sergipe, Brazil; ^6^Graduate Program in Health Sciences, Federal University of Sergipe, São Cristóvão, Sergipe, Brazil

## Abstract

**Background:**

Mortality in the intensive care unit (ICU) has been associated to an array of risk factors. Identification of risk factors potentially contribute to predict and reduce mortality rates in the ICU. The objectives of the study were to determine the prevalence and the factors associated with the mortality and to analyze the survival.

**Method:**

A cross-sectional study conducted in two clinical and surgical ICU in the state of Sergipe, northeastern Brazil. We enrolled 316 patients with at least 48 h of hospitalization, minimum age of 18 years old, sedated or weaned, with RASS ≥ −3, between July 2017 and April 2018. We categorized data in (1) age and gender, (2) clinical condition, and (3) prevalence of delirium. Data from enrolled patients were collected from enrollment until death or ICU discharge. Patients' outcomes were categorized in (1) death and (2) nondeath (discharge).

**Results:**

Twenty-one percent of participants died. Age (53 ± 17 years *vs*. 45 ± 18 years, *p* < 0.01), electrolyte disturbance (30.3% *vs* 18.1%, *p*=0.029), glycemic index (33.3% *vs* 18.2%, *p*=0.008), tube feeding (83.3% vs 67.1%, *p*=0.01), mechanical ventilation (50% *vs* 35.7%, *p*=0.035), sedation with fentanyl (24.2 *vs* 13.6, *p*=0.035), use of insulin (33.8% *vs* 21.7%, *p*=0.042), and higher Charlson score (2.61 *vs* 2.17, *p*=0.041) were significantly associated with death on the adjusted model. However, the regression model indicated that patients admitted from the emergency (HR = 0.40, *p*=0.006) and glycemic index alterations (HR = 1.68, *p*=0.047) were associated with mortality. There was no statistically significant difference (*p*=0.540) in survival between patients with and without delirium, based on the survival analysis and length of hospitalization.

**Conclusion:**

The prevalence of death was 21%, and age, electrolyte disturbance, glycemic index, tube feeding, mechanical ventilation, sedation with fentanyl, use of insulin, and higher Charlson score were associated with mortality.

## 1. Introduction

Patients with life-threatening conditions are treated in the intensive care unit (ICU). Treatment success and mortality rates in the ICU depend on the adequate utilization of human and technological sources [1]. ICU mortality has been associated with the length of hospitalization, patients' clinical condition, immobility [2], sedation, neurological disease, agitation, coma, intubation [3], mechanic ventilation, use of vasopressors drugs [4], glycemic index [5], sociodemographic characteristics, and delirium [6]. Prolonged ICU hospitalization seems to double the risk of death. However, about 47% of ICU patients die within 48 hours of admission [7]. A multicountry study identified that the majority of people in the ICU come from emergency wards and need mechanical ventilation, vasopressor medication, and hemodialysis [8]. In Brazil, ICU hospitalization encompasses people with cardiovascular, neurological diseases, sepsis, and accidents. The ICU mortality rates for the ICU patients reach 18% in Brazil and are frequently observed in public hospitals [9]. Sociodemographic changes impact ICU mortality with aging and associated comorbidities increasing the risk of death [2].

Thus, providing the best care encompasses the appropriate assessment of patients' condition. Selecting essential instruments to evaluate patients and determine standards of care is vital to ensure the quality of healthcare in the ICU. Several surveys can be employed to assess comorbidities and estimate mortality risk in this context [1, 10]. For instance, the Charlson comorbidity index (CCI) identifies potential risk factors and contributes to a safe healthcare plan [11]. In the intensive care environment, it is essential to accurately identify patient's characteristics, provide the best care, detect and prevent modifiable risk factors, and reduce mortality risk [12]. Instruments evaluating neurological conditions, such as the RASS (Richmond Agitation-Sedation Scale) and the Glasgow Coma Scale (GCS), contribute to a proper patient assessment and determination of care.

Here, we determine the prevalence and the factors associated with the mortality and analyze the survival in the ICU patients in Sergipe, northeastern Brazil. This region has a low human development index (HDI 0.665) and a population of approximately 2.3 million inhabitants [13]. Chronic degenerative disorders, automobilist accidents, homicide, suicide, and violence are the leading causes of death in Sergipe [14].

## 2. Materials and Methods

### 2.1. Design

We conducted a cross-sectional study in clinical and surgical intensive care units of the Unified Health System (SUS) in Sergipe, northeastern Brazil. This study is part of a project entitled “Incidence and risk factors for delirium and pharmacovigilance in the management of patients admitted to intensive care units.”

### 2.2. Participants

ICU patients admitted between August 2017 and October 2018 were considered for the study. We included patients aged 18 years or older, with at least 48 hours of ICU hospitalization, sedated or not, and the RASS score higher than −3. We excluded critically ill patients with a Glasgow score ≤8, aphasia, brain death, or under correctional custody.

### 2.3. Procedures and Measurements

Data were collected daily in the ICU. Time of the data collection varied according to the research team's availability. We retrieved sociodemographic data and clinical status and identified the incidence of delirium. We included admission information (from the inpatient or emergency wards), diagnosis at ICU admission, type of hospitalization (clinical or surgical), presence of pressure ulcers, and clinical manifestation of dehydration, fluid balance, diuresis, skin conditions [15], glycemic index (hypoglycemic ˂ 70 mg/dl or hyperglycemic ˃ 140 mg/dl) [16], and hypothermia (axillar temperature < 35°C) [15], and use of physical restraints, tube feeding, and mechanical ventilation.

We used the Richmond Agitation-Sedation Scale (RASS) and the Glasgow Coma Scale to evaluate consciousness. The Richmond Agitation-Sedation Scale (RASS) [17] was used to assess patients' levels of sedation. RASS is a 10-item numeral scale ranging from −5 (unarousable sedation, nonresponsive to verbal or physical stimulation) to +4 (combative). The Glasgow Coma Scale [18] is an easy-to-use scale, considered the gold-standard to measure the depth and duration of coma and impaired consciousness. Assessment of the level of consciousness includes evaluating ocular, verbal, and motor responses. The sum score classifies trauma as mild (GCS 13–15), moderate (GCS 9–12), or severe GCS ≤ 8.

The confusion assessment method in an intensive care unit (CAM-ICU) [19–21] was used to identify the occurrence of delirium, whereas the Charlson comorbidity index (CCI) was employed to identify clinical conditions that may influence the risk of death. The CCI is a 17-item scale with a score ranging from zero to six points. Higher scores indicate a higher risk of death [11, 22].

All participants were followed daily until ICU discharge or death. Demographic and clinical data were retrieved from medical records. The analysis divided patients into two groups according to hospitalization outcome as [1] death and [2] survival. Data were collected in two steps as [1] assessment with GCS or RASS and [2] assessment with CAM-ICU.

### 2.4. Data Processing and Analysis

Categorical variables were described utilizing absolute and relative frequencies. The continuous variables were described as by means and standard deviation. The associations between categorical variables were tested using Fisher's exact test, Pearson's chi-square, and Pearson chi-square with Monte–Carlo simulations. The adherence to normal distribution was tested by the Shapiro–Wilk method. Differences in central trend measurements were tested using the *t*-test for independent samples or the Mann–Whitney test. Prevalence ratios were estimated and adjusted by log-binomial regression and the backward selection method of selection of variables with input significance of 10% or 20%. The survival curve was estimated using the Kaplan–Meier estimator and the risk ratios through Cox regression. In all analyses, only valid observations were considered. The significance level adopted was 5% using the *R* core team in 2019.

### 2.5. Ethics Considerations

This study was approved by the Committee of Ethics in Research of the Federal University of Sergipe (Sergipe, Brazil, under the number: 2.051.128) on May 8^th^, 2017, and conducted under the Helsinki declaration.

## 3. Results

Eight hundred and thirty-five potential participants were screened from August 2017 to October 2018, and 316 patients hospitalized in two clinical and surgical ICU units in the Hospital de Urgência de Sergipe (HUSE) were included in the sample. Regarding hospitalization outcomes, 21% died. Compared to the patients who survived, participants in the group with death as outcome were older (53 ± 17 years *vs.* 45 ± 18 years, *p* < 0.01), mostly transferred from inpatient units with a diagnosis of sepsis (41.9% vs. 22.8%, *p*=0.003), and manifested delirium in the ICU (54.5% *vs.* 43.6%, *p*=0.112). Additionally, this group exhibited significant electrolyte disorders (30.3% *vs* 18.1%, *p*=0.029) and changes in glycemic index (33.3% *vs* 18.2%, *p*=0.008) were placed under tube feeding (83.3% *vs* 67.1%, *p*=0.01), mechanical ventilation (50% *vs* 35.7%, *p*=0.035), sedation with fentanyl (24.2 *vs* 13.6, *p*=0.035), and the insulin therapy (33.8% *vs* 21.7%, *p*=0.042) and had a higher Charlson score (2.3 ± 2.61 *vs* 1.6 ± 2.17, *p*=0.041) (Table 1).

Fourteen potential predictors of mortality were included in the logistic regression model (age, delirium, type of hospitalization, dehydration, electrolyte disturbances, changes in glycemic index, hypothermia, physical restraint, tube feeding, mechanic ventilation, pressure ulcers, use of anticonvulsant, insulin, and Charlson score). The adjusted model indicated that age, dehydration, tube feeding, and the use of anticonvulsant drugs increase the prevalence of death (Table 2).

**Figure 1:**
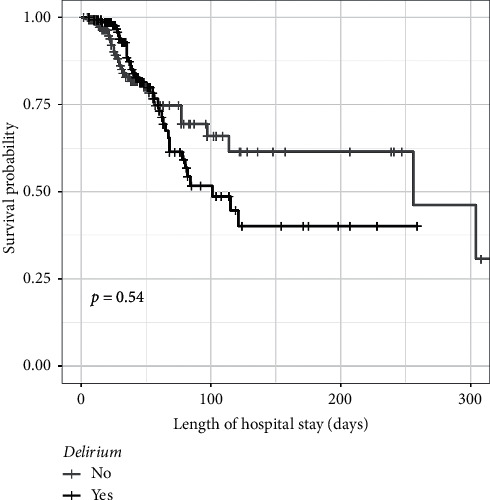
Survival curve among patients with and without delirium in the ICU.

According to the survival analysis curve (Figure 1), patients with delirium had a nonstatistically significant difference in the survival time (*p*=0.540) associated with the length of hospitalization with a mean survival time of 161.51 (95% CI: 126.65–196.36) days against 200.33 (95% CI: 166.49–234.17) days in patients without delirium.

## 4. Discussion

This study identified a 21% ICU mortality rate among patients in the hospital of Sergipe, northeastern Brazil. The mortality rate is considered high when compared to other national and international studies [1, 2, 23–25]. Age, admission from inpatient unit, sepsis, hydroelectrolyte imbalance, changes in glycemic index, tube feeding, mechanic ventilation, sedation with fentanyl, insulin use, and higher Charlson scores were associated with mortality in this study.

Early onset of chronic noncommunicable diseases among the population, associated with comorbidities, increase health system demand. Thus, countries lacking an effective health models in primary and secondary care almost always overload the tertiary sector with hospitalizations. This problem contributes to increased mortality particularly among individuals in critical condition [25, 26].

The adjusted model described the association between older age and prevalence of mortality. Other studies identified similar relationship in ICU patients; however, the risk of death was linked to elderly patients [1, 2, 23, 25, 27]. Incidence of chronic diseases among younger adults has been observed in Brazil. Particularly, hypertension has been prevalent among individuals with a mean age 44, whereas the mean age for diabetes is 49 years old [28]. Similar to other studies, we observed higher mortality among male patients (62.1%) [22–24], inpatients transferred to the ICU (18.2%), and diagnosed with sepsis (41.9%). Those findings are 2.5 times higher than those observed in multicentric studies conducted in 65 ICU from all Brazilian territory where mortality rates by sepsis alone were 16.7% [29]. The higher incidence of sepsis in the ICU can be explained by patients' fragile clinical condition, emphasized by higher Charlson score, and constant need for invasive procedures and antibiotics that ultimately jeopardize natural immune response and cultiminate in death [29, 30]. The type of care before ICU admission was mentioned by other researchers as potentially increasing the risk of death, and as once transferred to the ICU, individuals required critical hemodynamic care and constant surveillance [23].

Dehydration, tube feeding, and use of anticonvulsant drugs were associated with mortality in this study. Enteral nutrition is a common standard of care for critical patients [31]. Research indicates that a regular nutritional evaluation using antropometric and the biochemical analysis are complicated in critical patients due to weight loss, dehydration, and edema [31]. Additionally, the pharmacological therapy with opioids, benzodiazepnics, and insulin for the treatment of physiological changes (pain, agitation, anxiety, and metabolic disorders) and to minimize external factors such as noise, often contribute to higher mortality rates in the ICU [32].

Mechanic ventilation and consequent higher incidence of adverse events such as infection and pneumonia [6] were identified as a predictor of mortality in this study [6, 33]. Changes in glycemic index are often observed in critically ill patients as a consequence of corticoid use and enteral and parental nutrition [34]. Hyperglicemia and oxidative stress jeopardize the organic function [35] and have been associated with 14.7% of death among critically ill patients [36]. Both hyper and hypoglycemic states have been associated with the increased need for dialysis and mechanic ventilation [37]. An accurate monitoring of glycemic index is crucial for ICU patients [38, 39].

Contrarily to previously published studies [6, 40], delirium was not statistically associated with the outcomes of death or survival in our study. Although more patients with delirium died, the survival curve indicated that the death was associated with longer hospitalization and 200 days of survival. Evidence suggests a 27.8% mortality rate for ICU patients, and those without delirium are 1.5 times more likely to die. Other studies indicated that individuals with delirium are 1.7 times more likely to die [6].

Death in the critical care unit is a multifactorial phenomenon. Thus, identifying the modifiable factors such as duration of sedation, early mobilization, and weaning from mechanical ventilation potentially contribute to better ICU outcomes.

Our study has several limitations. First, the cross-sectional method does not allow for causality inference in terms of delirium and mortality. Second, we conducted a single-sited study, which could induce bias in recruitment and prevent generalization of findings. Further investigation is warranted expanding the study population and exploring other potential predictions and comorbidities that may increase the mortality risk in the ICU.

## 5. Conclusions

Modifiable factors associated with higher mortality in the ICU were age, admission from inpatient units, and diagnosis of sepsis. Additionally, hydroelectrolyte imbalance, changes in glycemic index, tube feeding, mechanical ventilation, sedation with fentanyl, use of insulin therapy, and higher Charlson scores were correlated with mortality. The adjusted model indicated that age, dehydration, tube feeding, and use of anticonvulsant drugs increased the prevalence of death. Patients with delirium who died represented 54.5% of the sample; however, the survival analysis indicated that the length of hospitalization but not delirium was associated with mortality.

## Figures and Tables

**Table 1:** Factors associated with hospital death.

Variables		Death (*n* = 66)	Discharge (*n* = 250)	*p* value
Age in years, mean (SD)		53 (17)	45 (18)	0.001^*W*^
Delirium, *n* (%)				
Yes		36 (54.5)	109 (43.6)	0.112^*Q*^
No		30 (45.5)	141 (56.4)	
Gender, *n* (%)				
Female		25 (37.9)	81 (32.4)	0.402^*Q*^
Male		41 (62.1)	169 (67.6)	
Original admission, *n* (%)				
Inpatient unit		12 (18.2)	14 (5.6)	0.012^*QM*^
Clinical category, *n* (%)				
Sepsis		18 (41.9)	28 (22.8)	0.003^*QM*^
Hydroelectrolytic disorder, *n* (%)		20 (30.3)	45 (18.1)	0.029^*Q*^
Glycemic index, *n* (%)		22 (33.3)	45 (18.2)	0.008^*Q*^
Immobility, *n* (%)		12 (18.5)	50 (20.3)	0.862^*F*^
Physical containment, *n* (%)		45 (68.2)	142 (57)	0.101^*Q*^
Tube feed, *n* (%)		55 (83.3)	165 (67.1)	0.010^*F*^
Mechanical ventilation, *n* (%)		33 (50)	89 (35.7)	0.035^*Q*^
Wound injury, *n* (%)		22 (34.4)	56 (23.2)	0.069^*Q*^
Fentanyl use, *n* (%)		16 (24.2)	34 (13.6)	0.035^*F*^
Insulin use, *n* (%)		22 (33.8)	54 (21.7)	0.042^*Q*^
Charlson score total with Adjustment, mean (SD)		2.3 (2.61)	1.62 (2.17)	0.041^*W*^

SD, standard deviation. *n*, absolute frequency. %, relative percentage frequency. ^*F*^Fisher's exact test. ^*Q*^Pearson chi-square test. ^*QM*^Pearson chi-square test with Monte–Carlo simulations. ^*W*^Mann–Whitney test.

**Table 2:** Prevalence ratios for death.

	Death		
	PR (CI 95%)	PRa (CI 95%)	*p* value
Age	1.02 (1.01–1.03)	1.02 (1.01–1.03)	0.009
CAM			
Delirium	1.41 (0.92–2.18)		
No delirium	1		
Hospitalization type			
Clinical	1.53 (0.42–5.57)		
Dehydration	2.20 (1.03–4.72)	2.51 (1.55–4.08)	<0.001
Hydroelectrolytic disorder	1.67 (1.07–2.62)		
Changes in glycemic index	1.84 (1.19–2.83)		
Hypothermia	1.85 (0.97–3.49)		
Physical containment	1.47 (0.92–2.34)		
Tube feed	2.09 (1.15–3.81)	1.85 (1.01–3.37)	0.045
Mechanical ventilation use	1.58 (1.03–2.42)		
Wound injury	1.52 (0.97–2.38)		
Anticonvulsant use	0.61 (0.34–1.07)	0.51 (0.27–0.96)	0.036
Insulin use	1.60 (1.03–2.50)		
Charlson score	1.09 (1.01–1.18)		

PR, prevalence ratio. PRa, adjusted prevalence ratio. CI 95%, 95% confidence interval. The multifactorial analysis using the Cox regression model indicated that patients admitted from the emergency (HR = 0.40, *p*=0.006) and patients with an altered glycemic index (HR = 1.68, *p*=0.047) were more likely to die in the ICU (Table 3).

**Table 3:** Cox model for the risk of death and clinical variables.

	Death	*p* value
	HR (CI 95%)	
CAM-ICU		
Delirium	1.17 (0.71–1.91)	0.537
No delirium	1	
Origin		
Inpatient unit	1	
Emergency room	0.40 (0.21–0.76)	0.006
Hydroelectrolytic disorder	1.62 (0.96–2.75)	0.073
Changes in glycemic index	1.68 (1.01–2.81)	0.047
Hypoxemia	2.50 (1.00–6.29)	0.051
Anticonvulsive use	0.57 (0.31–1.07)	0.081

HR, hazard risk. CI 95%, 95% confidence interval.

## Data Availability

The data used to support the findings of this study are available from the corresponding author upon request.
